# Contractility of Induced Pluripotent Stem Cell-Cardiomyocytes With an *MYH6* Head Domain Variant Associated With Hypoplastic Left Heart Syndrome

**DOI:** 10.3389/fcell.2020.00440

**Published:** 2020-06-23

**Authors:** Min-Su Kim, Brandon Fleres, Jerrell Lovett, Melissa Anfinson, Sai Suma K. Samudrala, Lauren J. Kelly, Laura E. Teigen, Matthew Cavanaugh, Maribel Marquez, Aron M. Geurts, John W. Lough, Michael E. Mitchell, Robert H. Fitts, Aoy Tomita-Mitchell

**Affiliations:** ^1^Division of Pediatric Cardiothoracic Surgery, Department of Surgery, Medical College of Wisconsin, Herma Heart Institute, Milwaukee, WI, United States; ^2^Department of Biological Sciences, Marquette University, Milwaukee, WI, United States; ^3^Department of Cell Biology, Neurobiology and Anatomy, Medical College of Wisconsin, Milwaukee, WI, United States; ^4^Department of Physiology, Medical College of Wisconsin, Milwaukee, WI, United States; ^5^Department of Biomedical Engineering, Medical College of Wisconsin, Milwaukee, WI, United States

**Keywords:** HLHS, hypoplastic left heart syndrome, iPSC-cardiomyocytes, *MYH6*, contractility, CRISPR/Cas9, sarcomere

## Abstract

Hypoplastic left heart syndrome (HLHS) is a clinically and anatomically severe form of congenital heart disease; however, its etiology remains largely unknown. We previously demonstrated that genetic variants in the *MYH6* gene are significantly associated with HLHS. Additionally, induced pluripotent stem cell-derived cardiomyocytes (iPSC-CMs) from an HLHS-affected family trio (affected parent, unaffected parent, affected proband) carrying an *MYH*6-R443P head domain variant demonstrated dysmorphic sarcomere structure and increased compensatory *MYH7* expression. Analysis of iPSC-CMs derived from the HLHS trio revealed that only beta myosin heavy chain expression was observed in CMs carrying the *MYH6*-R443P variant after differentiation day 15 (D15). Functional assessments performed between D20-D23 revealed that *MYH6*-R443P variant CMs contracted more slowly (40 ± 2 vs. 47 ± 2 contractions/min, *P* < 0.05), shortened less (5.6 ± 0.5 vs. 8.1 ± 0.7% of cell length, *P* < 0.05), and exhibited slower shortening rates (19.9 ± 1.7 vs. 28.1 ± 2.5 μm/s, *P* < 0.05) and relaxation rates (11.0 ± 0.9 vs. 19.7 ± 2.0 μm/s, *P* < 0.05). Treatment with isoproterenol had no effect on iPSC-CM mechanics. Using CRISPR/Cas9 gene editing technology, introduction of the R443P variant into the unaffected parent’s iPSCs recapitulated the phenotype of the proband’s iPSC-CMs, and conversely, correction of the R443P variant in the proband’s iPSCs rescued the cardiomyogenic differentiation, sarcomere organization, slower contraction (*P* < 0.05) and decreased velocity phenotypes (*P* < 0.0001). This is the first report to identify that cardiac tissues from HLHS patients with *MYH6* variants can exhibit sarcomere disorganization in atrial but not ventricular tissues. This new discovery was not unexpected, since *MYH6* is expressed predominantly in the postnatal atria in humans. These findings demonstrate the feasibility of employing patient-derived iPSC-CMs, in combination with patient cardiac tissues, to gain mechanistic insight into how genetic variants can lead to HLHS. Results from this study suggest that decreased contractility of CMs due to sarcomere disorganization in the atria may effect hemodynamic changes preventing development of a normal left ventricle.

## Introduction

Hypoplastic left heart syndrome (HLHS) is a severe form of congenital heart disease (CHD) characterized by atresia/stenosis of the aortic and mitral valves, and severe hypoplasia of the left ventricle and aorta (Noonan and Nadas, 1958). HLHS affects more than one in every 4000 live births (Mai et al., 2019). Evidence supporting a genetic basis for HLHS includes observations of familial clustering, high heritability, and its occurrence with specific chromosomal disorders such as Turner and Jacobsen syndromes (Tomita-Mitchell et al., 2016; Lara et al., 2017; Yagi et al., 2018). HLHS exhibits complex genetic inheritance along with an increased frequency of bicuspid aortic valve (BAV) and coarctation of the aorta (CoA) in relatives of HLHS patients (Hinton et al., 2007, 2009). Variants in genes such as *GJA1* (Dasgupta et al., 2001), *NKX2.5* (Elliott et al., 2003), *NOTCH1* (Garg et al., 2005; McBride et al., 2008; Hrstka et al., 2017; Yang et al., 2017), and *MYH6* (Theis et al., 2015; Tomita-Mitchell et al., 2016), as well as observations of syndromic or rare copy number variants (CNVs) in cardiomyogenic genes (Grossfeld, 2007; Grossfeld et al., 2009; Tomita-Mitchell et al., 2012; Warburton et al., 2014; Glidewell et al., 2015) have been associated with HLHS. We previously reported that rare variants in *MYH6* (alpha myosin heavy chain; α-MHC) were observed in 10% of HLHS patients, and that cardiac transplant-free survival was reduced in HLHS subjects containing *MYH6* variants in comparison with HLHS patients without *MYH6* variants. Furthermore, cardiac tissue from *MYH6* variant carriers exhibited significant upregulation of sarcomere genes, including *ACTA1* (actin alpha 1), *MYL2* (myosin light chain 2), *TNNT2* (cardiac troponin T), and the *MYH6* homolog *MYH7*, which encodes the beta-myosin heavy chain (β-MHC) isoform. Using cardiomyocytes (CMs) derived from induced pluripotent stem cells (iPSCs) obtained from an HLHS patient carrying an *MYH6*-R443P variant, we discovered that sarcomere structure was dysmorphic (Tomita-Mitchell et al., 2016).

Two myosin heavy chain proteins (α-MHC and β-MHC) are expressed in the human heart. During development, α-MHC is expressed in both the primitive atrium and ventricle until gestational day 35, after which its expression continually decreases in the ventricle (Wessels et al., 1990, 1991). In contrast, β-MHC expression gradually increases in the ventricles throughout gestation and this expression pattern persists throughout adult stages, so that β-MHC is the predominant ventricular isoform, while α-MHC is the predominant atrial isoform in adults (Cummins and Lambert, 1986; Reiser et al., 2001). We previously noted that *MYH6* mRNA strongly predominates during the earliest stages of *in vitro* cardiomyogenesis in H1 human embryonic stem cells wherein *MYH6* comprises ∼99% of total MHC transcripts in differentiation day 8 (D8) cultures, and declines to ∼86% at D14 (Kim et al., 2015). This likely reflects a prominent role for α-MHC in nascent myocyte development that may be disrupted by *MYH6* variants associated with HLHS.

Fetal heart development relies on proper blood flow, as signaling pathways responsive to shear stress and pressure-related strain both affect cardiac chamber formation. The prevailing hypothesis is that HLHS pathophysiology stems from impaired blood flow through the left ventricle (LV) during cardiogenesis (Fishman et al., 1978; Gruber and Epstein, 2004; deAlmeida et al., 2007). Our findings are consistent with this, as disruptions in an atrial protein such as α-MHC would alter ventricular preload with consequent defective expansion and/or differentiation of cardiomyocytes resulting in a dysmorphic and dysfunctional ventricle (Hove et al., 2003; Burggren et al., 2004; Hierck et al., 2008; McCain and Parker, 2011; Santhanakrishnan and Miller, 2011; Lee et al., 2016; McCormick and Tzima, 2016; Hoog et al., 2018). This is further supported by studies of weak atrium (*wea*) zebrafish, which harbor *myh6* mutations and exhibit defects in both cardiac chambers, including defective atrial contraction along with abnormal sarcomere organization and an underdeveloped ventricle (Berdougo et al., 2003).

Current non-invasive imaging methods allow detection of HLHS as early as 16 weeks gestational age (Friedberg et al., 2009; Galindo et al., 2009), long after the fetal heart is formed and septated at 7–8 weeks. The primitive heart, expressing only *MYH6*, begins beating even earlier, during the third week of development. Thus, the pathological changes associated with HLHS are likely present long before we are able to detect them. Given these limitations, developmental defects such as HLHS are often investigated using murine models. However, the chamber-specific MHC expression in rodents is the opposite of humans, making them an unsuitable option for our studies. Here we studied human cardiac tissue and patient-specific iPSC-CMs to identify structural characteristics of atrial and ventricular tissue of HLHS patients and contractile properties of human iPSC-CMs with an *MYH6* variant. The latter permitted us to monitor early stages of cardiomyogenesis in CMs of HLHS patients *in vitro*. Our hypothesis is that the *MYH6* variant iPSC-CMs exhibit depressed extent and velocity of shortening that can be rescued by correcting the variant in proband-iPSCs using Clustered Regularly Interspaced Short Palindromic Repeats (CRISPR)/Cas9 gene editing. Importantly, we show the feasibility of employing iPSC-CMs to ascertain functional consequences of the *MYH6*-R443P variant, demonstrating that changes in the myosin isoforms confer contractile limitations.

## Materials and Methods

### Study Participants

Study subjects have been previously described (Tomita-Mitchell et al., 2016). All subjects provided written informed consent, and procedures were approved by the MCW Institutional Review Board and conformed to the principles in the Declaration of Helsinki.

### Gene Editing iPSC Lines

#### Guide RNA and ssODN Design

All CRISPRs were designed as previously described (Mitzelfelt et al., 2017). Briefly, CRISPR target sites within 30 bps of the single nucleotide polymorphism (SNP) *MYH6*-R443P were identified using ZiFiT Targeter (Version 4.2). Three guide RNAs were cloned into pX330-U6-Chimeric_BB-CBh-hSpCas9 (Addgene, Cambridge, MA, United States) to introduce *MYH6*-R443P into the heart healthy parent’s iPSCs. CRISPR efficiency was determined using the Cel-1 surveyor assay in HEK293 cells. The variant *MYH6*-R443P was flanked approximately 60 bp on either side by single strand oligonucleotides (ssODNs) (Table 1). Silent mutations were incorporated to prevent re-cutting by Cas9 by disrupting the target sequence. An additional AAVS1 locus was targeted for incorporation of GFP-puromycin resistance using the AAVS1 Safe Harbor TALE-Nuclease kit (SBI, Palo Alto, CA, United States).

**TABLE 1:** Oligonucleotides used for gene editing, sequencing, and genotyping.

**CRISPR guide RNAs**	
*MYH6*-R443P introduction	tgagaagatgttcaactgga
*MYH6*-R443P correction	gacgcccatcaacgccaccc
*AAVS1* locus	gtcaccaatcctgtccctag
**ssODNs**	
*MYH6*-R443P introduction	ccagcgatgtccaggactcctatgaagtactggcgtggctgcttgg tctccagggtggcgttgatgggcgtcaccatccaattgaacatcttc tcatacactgccttggccagagccccgatggagtagtacacctgc tgc
*MYH6*-R443P correction	ctctggccaaggcagtgtatgagaagatgttcaactggatggtgac gcgcatcaacgcgacgctggagaccaagcagccacgccagtac ttcataggagtcctggacatcgctggcttcg
**PCR primers for genotyping (5′∼3′)**	
F-*MYH6*-R443P locus	ggcaacgagtatgtcaccaa
R-*MYH6-*R443P locus	ctttgtctggatggcagagg
**Allele-specific SNP genotyping (5′∼3′)**	
Reference probe	FAM-tggtgacgcgcat
Variant probe	VIC-atggtgacgcccat
F-*MYH6*-R443P locus	aaggcagtgtatgagaagatgttca
R-*MYH6-*R443P locus	aagtactggcgtggctgctt

To correct the *MYH6*-R443P variant in proband iPSCs, we designed CRISPR targets (Table 1) to align with the variant allele and purchased synthesized RNA (AltR CRISPR-Cas9 cr RNA, Integrated DNA Technologies, Coralville, IA, United States), RFP-labeled repetitive sequence (AltR CRISPR-Cas9 tracr RNA, Integrated DNA Technologies, Coralville, IA, United States) and purified Cas9 enzyme (40 Um/U1). Additionally, we designed and validated a CRISPR targeting the AAVS1 locus to introduce a puromycin resistance cassette (Integrated DNA Technologies, Coralville, IA, United States).

#### Transfecting iPSCs

Prior to transfection, iPSCs were pretreated for 3–4 h with 5 μM Rho-associated kinase (ROCK) inhibitor (Y27632, Stemgent, Lexington, MA, United States), dissociated with accutase (Thermo Fisher Scientific, Waltham, MA, United States) to ensure single cell suspension, and transferred to an electroporation cuvette. For each transfection (D0), 1 μg of the gene-specific CRISPRs, 80 μM of the relevant ssODN, 1 μg of each of the two AAVS1-specific cassettes, and 1 μg AAVS1 donor plasmid were added to 100 μl P4 solution (Lonza, Basel, Switzerland) and electroporated using program CB-150 on a 4D Nucleofector^TM^ (Lonza, Basel, Switzerland) into iPSCs (1 × 10^6^ cells/transfection). iPSCs from each transfection were then seeded into one well of a geltrex-coated 24-well plate (5 × 10^5^ cells/cm^2^) for recovery in mTeSR1 supplemented with 5 μM ROCK inhibitor. The following day (D1), iPSCs were cultured with mTeSR1. Two days post-transfection (D2), iPSCs from one well of 24-well plate were sub-cultured at a clonal density (∼5 × 10^4^ cells/cm^2^) into all wells of a 6-well plate pre-seeded with mitomycin C-treated mouse embryonic fibroblast (MEF) feeder cells (Thermo Fisher Scientific, Waltham, MA, United States) in human ESC medium. ESC medium was comprised of Knockout DMEM (Thermo Fisher Scientific, Waltham, MA, United States) supplemented with 20% Knockout Serum Replacement (Thermo Fisher Scientific, Waltham, MA, United States), MEM-NEAA (Thermo Fisher Scientific, Waltham, MA, United States), 2 mM L-glutamine (Thermo Fisher Scientific, Waltham, MA, United States), Penicillin/Streptomycin, 0.1 mM β-mercaptoethanol (Sigma-Aldrich, St. Louis, MO, United States), 10 ng/ml human basic fibroblast growth factor (bFGF, Cell Signaling, Danvers, MA, United States), 50 ng/ml L-ascorbic acid (Sigma-Aldrich, St. Louis, MO, United States) and 5 μM ROCK inhibitor. Medium was changed 2 days later (D4) to ESC medium without ROCK inhibitor. Five days post-seeding (D5), medium was changed to puromycin (0.5 g/ml)-supplemented MEF-conditioned medium with bFGF and L-ascorbic acid, which was replaced every 2 days for 1 week. Following maintenance (∼D12), distinct colonies (∼1 mm diameter) were manually transferred to a single well of a 24-well plate pre-coated with geltrex in mTeSR1 medium plus ROCK inhibitor without MEFs. Following genotyping (see below), clones were expanded to a geltrex-coated well of a 12-well plate in mTeSR1 plus ROCK inhibitor and further expanded and frozen in mFreSR freezing medium (STEMCELL Technologies, Vancouver, BC, Canada). Isolated CRISPRed iPSC lines were frequently sub-cloned to ensure homogeneity of the population.

#### Allele-Specific SNP Genotyping and Sanger Sequencing

To isolate genomic DNA from iPSCs, 30 μl Quick Extract Solution (Epicentre, Madison, WI, United States) was added to each cell pellet and incubated for 15 min at 65°C, followed by 5 min at 95°C. PCR was carried out using gene-specific primers (Table 1), and 2 μl of PCR product were used for further allele-specific SNP genotyping with Quantstudio7 (Thermo Fisher Scientific, Waltham, MA, United States). After confirmation of gene edit, ∼100 ng of PCR product was treated with Exosap-IT (Thermo Fisher Scientific, Waltham, MA, United States) and sent to Retrogen (San Diego, CA, United States) for Sanger sequencing. Clones that were positive for iPSCs containing the edited *MYH6*-R443P gene were sequenced at the conserved region in *MYH7* as well to check the CRISPR’s off-target activity.

### Cardiomyocyte Differentiation of iPSCs

Induced pluripotent stem cell (iPSC) lines were generated from dermal fibroblasts donated by HLHS probands and their parents. Fibroblasts were reprogrammed to pluripotent stem cells using Sendai reprogramming as previously described (Tomita-Mitchell et al., 2016). Pluripotency was confirmed with morphological appearance and % of cells exhibiting Oct4-positive immunostaining (99–100%). The cells were karyotypically normal and had the ability to differentiate into multiple lineages (definitive endoderm and cardiomyogenic mesoderm) (Tomita-Mitchell et al., 2016). In short, iPSCs were maintained in a hypoxic incubator at 5% CO_2_ and 4% O_2_. All experiments were performed on cells during passages 10–50. One day prior to inducing cardiomyocyte (CM) differentiation, the iPSCs were seeded at a cell density of >1 × 10^5^/cm^2^ on a 10 μg/cm^2^ geltrex-coated (Thermo Fisher Scientific, Waltham, MA, United States) tissue culture dish. The cells were cultured in mTeSR1 medium (STEMCELL Technologies, Vancouver, BC, Canada) supplemented with 5 μM ROCK inhibitor. The cells were induced with 10 μM CHIR99021 (Stemgent, Lexington, MA, United States) and 10 ng/ml Activin-A (R&D Systems, Minneapolis, MN, United States) in insulin-free RPMI/B27 (Thermo Fisher Scientific, Waltham, MA, United States). On D3, the medium was exchanged for insulin-free RPMI/B27 with 5 μM IWP (Tocris, Bristol, United Kingdom). Starting at D7, at 2-day intervals the medium was changed to RPMI/B27 with insulin (Thermo Fisher Scientific, Waltham, MA, United States). Cultures were evaluated for % of cardiac troponin T (cTnT) positive cells via flow cytometry and MHC (MF20) expression by immunostaining. The cardiomyocytes were either frozen at D15, or were sub-cultured between D10–D15 after dissociation into single cells with 0.25% trypsin; specifically, cells centrifuged at 1,000 rpm for 5 min were either frozen at a density of >2 × 10^6^ in cryovials with 10% DMSO and 90% FBS, or were re-plated at a density of 2 × 10^4^–5 × 10^4^/cm^2^ on a geltrex-coated 12 cm^2^ round cover glass for single cell-based contractility studies, or at >5 × 10^4^/ cm^2^ for automated contractility video analysis (Pulse Video Analysis, Dana solutions, Palo Alto, CA, United States).

### Cardiomyocyte Sarcomere-Immunostaining

The iPSC-CMs and tissues were co-immunostained with anti-sarcomeric α-actinin (AF594) (Abcam, Cambridge, MA, United States) to image sarcomere organization, and anti-MHC monoclonal MF20 (AF488) (DSHB, University of Iowa, Iowa City, IA, United States) to inform myocyte identity. Sarcomeres in iPSC-CMs were visualized with a Nikon inverted fluorescent microscope at 400x.

### Contractility of iPSC-CMs

All the contractile experiments with iPSC-CMs were conducted in a non-biased blinded fashion. Contractile data were collected from iPSC-CMs at differentiation days D20, D27, D34, and D41. Experiments were conducted over 3 days such that D20 includes D20-D23, D27 (D27–D30), D34 (D34–D37), and D41 (D41–D43). The iPSC-CMs were transferred to a recording chamber containing 1 mM Ca^2+^ Tyrode’s solution (35°C) mounted on the stage of an inverted microscope (Nikon TE2000). Spontaneously contracting iPSCs were visualized at 40x and contractile events recorded using a MyoCam-S video 250 Hz camera and a CFA300 cell framing adapter (IonOptix, Boston, MA, United States). The left and right edges of iPSC-CMs were defined and contractions per minute (CPM), shortening (μm), % cell shortening, and rates of shortening and relaxation (μm/second) were measured using the IonWizard image detection program (IonOptix, Boston, MA, United States). Ten cells on a 12 cm^2^ round cover glass were evaluated for 10 contractions each. For a subset of D20 iPSC-CMs, the same parameters were determined for responses to 1 and 2 Hz stimulation or to 1 μM isoproterenol. As an alternative method, an automated contractility software^1^ (Dana solution, Palo Alto, CA, United States) (Maddah et al., 2014, 2015) was used to study videos of contracting iPSC-CMs.

### Action Potentials of iPSC-CMs

Action potentials of iPSC-CMs were obtained with microelectrodes (50–70 MΩ) filled with 2.7 M KCl and 10 mM HEPES and recorded at 35°C using Clampex9.2 software (Axon Instruments, Axoclamp-2A, San Jose, CA, United States). For each iPSC-CM measured, the lowest membrane potential (referred to as diastolic potential), threshold potential, peak spike potential, and action potential duration were averaged from 5 action potentials per cell. Action potential duration (APD_90_) was defined as the time (ms) from the start of the action potential until membrane potential returned to 10% of its peak height.

### Calcium Transients of iPSC-CMs

The iPSC-CMs were plated on an 18 mm^2^ round cover glass at D15. At D20, the iPSC-CMs were incubated for 45 min at 35°C in 0.5 mM Ca^2+^ Tyrode’s solution containing 2.5 μM of Fura-2 AM (Invitrogen, Carlsbad, CA, United States). Then, cover glasses were inserted into a quick-release chamber (RC-49MFS, Warner Instruments, Hamden, CT, United States) bathed in 1 mM Ca^2+^ Tyrode’s solution at 35°C. Whole cell Ca^2+^ transients were recorded using Fura-2 AM as described previously (Wang and Fitts, 2018). Briefly, Fura-2 AM was excited at 340 and 380 nm switching between wavelengths with a LAMBDA DG4 (Sutter Instrument, Novato, CA, United States) and the fluorescence emission measured at 510 nm. To determine the Ca^2+^ transient rate of rise and fall and the peak amplitude, the ratio was analyzed with IonWizard and data were expressed as ratio units (peak amplitude) and ratio units/s (rate of transient rise and fall). Ten Ca^2+^ transients were averaged for every iPSC-CM.

### Protein Expression Analysis of iPSC-CMs

#### Sample Preparation

Following contractile studies, iPSC-CMs were scraped from cover glasses into 1 mM Ca^2+^ Tyrode’s solution and centrifuged for 5 min at 16,100 *× g*. The pellet of iPSC-CMs was collected in 10 μl SDS sample buffer (10% SDS, 23 mM EDTA, 50% glycerol, 0.4% bromophenol blue, and 5.1% β-mercaptoethanol, pH 6.8) and stored at −80°C until studied.

#### Silver Staining

MHC composition was determined by silver staining 5% SDS-PAGE gels as previously described (Sundberg et al., 2018). Briefly, gels were run for 28–30 h at 4°C to separate α-MHC and β-MHC, and ImageJ (National Institutes of Health) was used to quantify protein expression. Samples were also run for 1 h at 150 V on 12% precast polyacrylamide gels using a Mini-PROTEAN Electrophoresis Cell (Bio-Rad, Hercules, CA, United States) and silver stained to examine lower molecular weight proteins.

#### Western Blotting

Diluted samples were run on a 4–15% gradient gel for 1 h at 150 V and transferred to a 0.2 μm nitrocellulose membrane using a Mini Trans-Blot cell (Bio-Rad, Hercules, CA, United States) at 100 V for 50 min. The gel was run in Tris-glycine running buffer and transferred with running buffer containing 20% methanol. Following transfer, membranes were blocked for 3 h in 5% non-fat dry milk in TBS-0.1% Tween pH 7.6 (TBST) and probed with primary antibodies overnight at 4°C. Primary antibodies for MHC (MF20, 1:800, DSHB), GAPDH (ab181602, 1:1,000, Abcam, Cambridge, United Kingdom), and ventricular myosin light chain-2 (MLC2v) (7C9, 1:2,000, Fisher Scientific, Hampton, NH, United States) were diluted in 5% non-fat dry milk in TBST. Then, the blots were incubated with horseradish peroxidase-conjugated secondary antibodies (sc-516132, 1:6,500, Santa Cruz Biotechnology; NBP2-30347H, 1:6,500, Novus Biologicals; G-21234, 1:1,600, Fisher Scientific, Hampton, NH, United States) in 5% non-fat dry milk in TBST for 1 h at room temperature. Protein expression was detected on autoradiography film (Hyblot CL, Fisher Scientific, Hampton, NH, United States) by chemiluminescence. ImageJ (National Institutes of Health) was used for densiometric analysis and protein expression was normalized to GAPDH.

### Tissue Sarcomere-Immunostaining

#### Tissue Sample Preparation

A total of 27 samples from atrial and ventricular tissues were examined, including two 4-day old neonatal atrial tissues, one from an HLHS subject without an *MYH6* variant and one from an HLHS subject with the *MYH6*-R443P variant. The 25 non-neonatal tissues were from subjects between 227 days to 24-years old. These samples were divided into three study groups: control (non-HLHS CHDs and structurally normal cardiac control), HLHS subjects without an *MYH6* variant, and HLHS subjects with an *MYH6* variant. In each study group, when available, we included atrial and ventricular tissues. In all cases, the samples were pieces of tissue dissected and phenotyped at the time of surgery. They were snap frozen immediately after removal from the patient. Some tissues came at the time of heart implantation, and some came from the time of reparative surgery.

#### Tissue Immunostaining

Cryo-sections (10 μm) of the frozen tissues were prepared. The tissues were co-immunostained with anti-sarcomeric α-actinin (AF594) (Abcam, Cambridge, MA, United States) to image sarcomere organization, and anti-MHC monoclonal MF20 (AF488) (DSHB, University of Iowa) to inform myocyte identity. Sarcomeres in tissue sections and iPSC-CMs were visualized with Structured Illumination Microscopy (SIM) at 1000x magnification.

### Statistical Analyses

#### Characterization of Gene-Edited iPSC-CMs

Significant differences between two groups were analyzed through an unpaired two-tailed Student’s *t*-test using Excel (Microsoft). If the data were not normally distributed, comparisons were made using the non-parametric Wilcoxon signed-rank test. Statistical significance was set at *P* < 0.05.

#### Contractile Studies

When necessary, data were transformed to meet assumptions of normality and homogeneity of variance. For the time course study, a two-way ANOVA was performed to compare iPSC-CM contractility of wild-type (WT) vs. variant (VAR; *MYH6*-R443P) at timepoints D20, D27, D34, and D41. *Post hoc* pair-wise comparisons were performed using Tukey’s test. All subsequent studies (action potentials, contractile responses to stimulation, isoproterenol, and Ca^2+^ transient recordings) were restricted to D20. Data were analyzed with a one-way ANOVA, except for stimulation data, which were analyzed with a two-way ANOVA. Statistics were performed using Minitab v18.1 with significance set at *P* < 0.05.

## Results

### CRISPR/Cas9 Gene Edited iPSC-CMs

#### Insertion of the *MYH6*-R443P Variant Into the Heart Healthy Parent’s iPSCs Results in Expression of the Proband’s Sarcomere Phenotype

**FIGURE 1:**
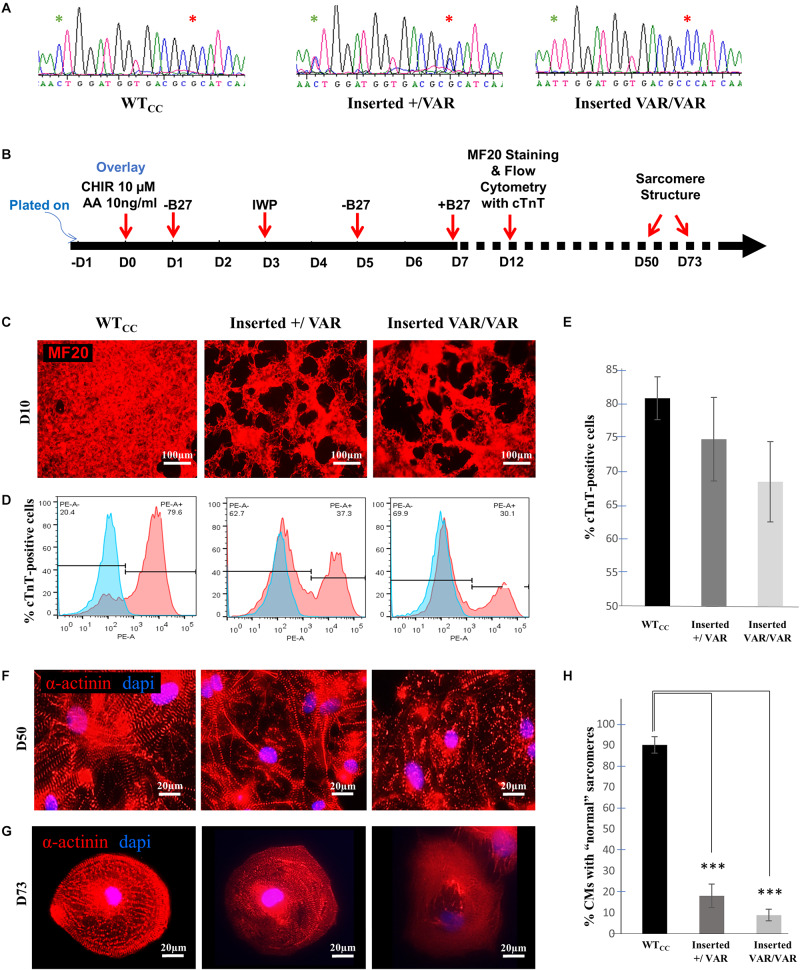
*MYH6*-R443P VAR-inserted iPSC-CMs recapitulate phenotypes of patient-specific iPSC-CMs. **(A)** Sequences at the *MYH6-*R443 locus in VAR-inserted lines. * is R443 locus and * is locus for silencing mutation. **(B)** Scheme for differentiating CMs using small molecule Gsk3 inhibitor (CHIR99021) with Activin-A and Wnt inhibitor (IWP). **(C)** Representative immunostaining of VAR-inserted iPSC-CMs with MF20 at D12. **(D)** Representative flow cytometry of cardiac troponin T (cTnT) positive cells in CRISPRed iPSC-CMs at D12. **(E)** Flow cytometry of cells cultured in parallel with those in **(D)**, showing % cTnT-positive cells in VAR-inserted iPSC-CMs at D10-15. Data were compiled from 16 replicates in each cell type and 5 separate experiments. **(F)** Sarcomeres are dysmorphic in VAR-introduced iPSC-CMs as compared to WT_CC_. α-actinin immunostaining (red) is seen in high density cultures at D50. **(G)** Comparative sarcomere organization in single cell iPSC-CMs isolated after sub-culturing at a low density at D69-D73. **(H)** % of cells with normal organized sarcomeres in both VAR-inserted iPSC-CMs, +/VAR and VAR/VAR in parallel to those in **(G)**. WTcc, CRISPRed control wild-type; +/VAR, *MYH6-*R443P heterozygous inserted; VAR/VAR, *MYH6-*R443P homozygous inserted. Values are means ± SE. Student’s *t*-test (two-tailed, equal variance), ***P < 0.0005.

Using CRISPR/Cas9 gene editing, the *MYH6*-R443P variant was introduced into the heart healthy parent’s iPSCs, generating CRISPRed control wild-type (WTcc) lines, as well as lines having one allele (inserted +/VAR) or both alleles inserted (inserted VAR/VAR). The CRISPR target site has one base-pair mismatch, and the sequences of both *MYH6* (Figure 1A) and its close homologue *MYH7* (Supplementary Figure S1) were confirmed in CRISPRed iPSCs with Sanger sequencing for any off-target effects. The experiments performed in VAR-inserted cells were obtained with only one line per genotype. Following cellular expansion and verification of genotype, iPSCs representing WTcc, inserted +/VAR and inserted VAR/VAR were induced to undergo cardiomyogenic differentiation as we previously described (Kim et al., 2015) (depicted in Figure 1B). The efficiency of cardiomyogenic differentiation during D10–D15 was reduced in both the inserted +/VAR and inserted VAR/VAR iPSC-CMs determined by MF20 immunostaining (Figure 1C) and flow cytometry assessment of % cTnT-positive cells (Figures 1D,E). We also evaluated sarcomere structure in iPSC-CMs at later stages of differentiation in both mass cultured CMs at D50 (Figure 1F) and individual CMs isolated from the culture at D73 (Figure 1G). Over 100 isolated iPSC-CMs of each line were judged for dysmorphic sarcomere organization at D69 and D73 (Figure 1H). “Normal” iPSC-CMs exhibit crisp and elongated ladders with wide Z-bands. Dysmorphic iPSC-CMs are characterized by more than 50% of the myocyte area displaying blurred α-actinin staining and/or sarcomeric ladders with punctate or truncated deposits of α-actinin (Figures 1G,H). iPSC-CMs with the *MYH6*-R443P variant were substantially disorganized in both the inserted +/VAR and inserted VAR/VAR compared to WTcc-CMs.

#### Correction of the *MYH6*-R443P Variant in iPSCs Normalizes the Proband’s Sarcomere Phenotype

**FIGURE 2:**
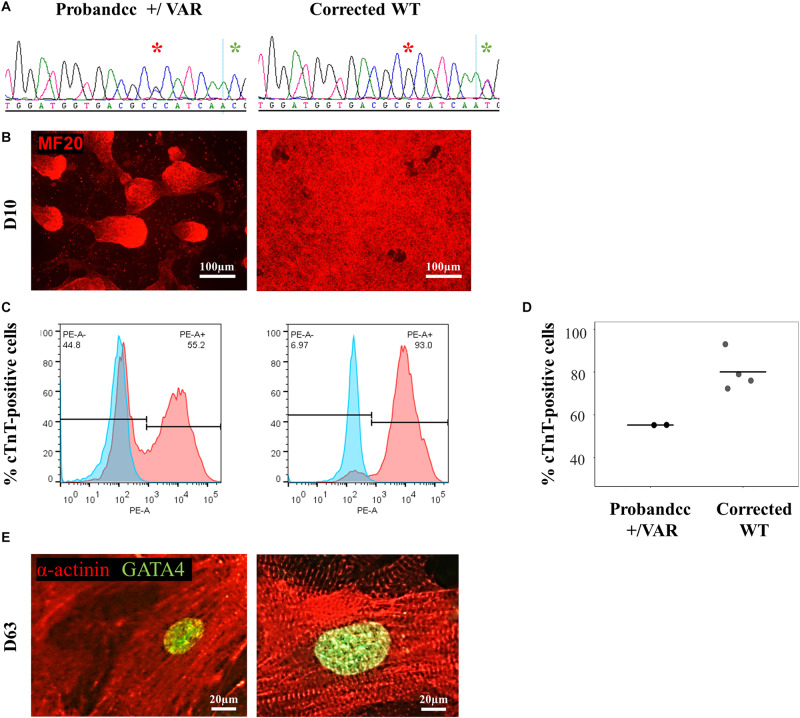
*MYH6*-R443P VAR-corrected iPSC-CMs rescues the phenotype of proband iPSC-CMs. **(A)** Sequences at the *MYH6-*R443P locus in corrected line. * is the R443 locus and * is the locus for a silencing mutation. **(B)** Representative immunostaining of VAR-corrected iPSC-CMs with MF20 (red) at D10. **(C)** Representative flow cytometry of cardiac troponin T (cTnT) positive cells in corrected iPSC-CMs at D12. **(D)** Flow cytometry of cells cultured in parallel with those in **(C)**, showing % cTnT-positive cells at D10. Each dot is a technical replicate (Probandcc *n* = 2 and corrected WT *n* = 4). **(E)** Sarcomeres appear more “normal” in corrected WT iPSC-CMs compared to Probandcc iPSC-CMs. α-actinin immunostaining is seen in red and GATA4 in green in high density cultures at D63. Probandcc, CRISPRed control proband; corrected WT, CRISPRed and corrected *MYH6*-R443P variant.

Using CRISPR-Cas9, we corrected the *MYH6*-R443P variant (corrected WT) in the proband’s iPSCs (Figure 2A). This improved the efficiency of cardiomyogenic differentiation as assessed by MF20 immunostaining (Figure 2B) and by flow cytometric quantitation of the % of cTnT-positive cells (Figures 2C,D). Perhaps most notable was the appearance of the sarcomeres, which were relatively disorganized in CRISPRed control proband (Probandcc) iPSC-CMs expressing the R443P variant (Figure 2E, left) and became organized upon correction in corrected WT iPSC-CMs (Figure 2E, right).

### Contractility of iPSC-CMs From WT, VAR, and *MYH6*-R443P CRISPRed Lines

**FIGURE 3:**
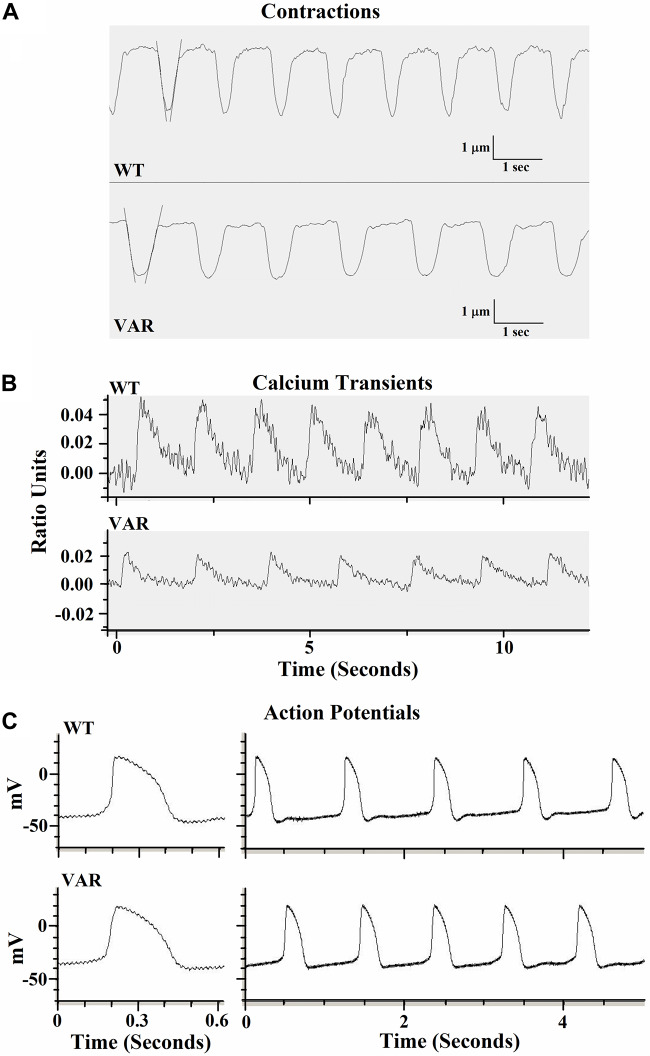
Representative traces of contractile properties of single iPSC-CMs at D20. **(A)** Spontaneously contracting WT and VAR single iPSC-CMs. Best-fit lines are shown to demonstrate the measurement of maximal cell shortening and relaxation rates (IonOptix). **(B)** Representative calcium transients of WT and VAR single iPSC-CMs **(C)** Representative action potentials of WT and VAR single iPSC-CMs, followed by a 5 s recording from the same cell. CPM, contraction per minute; WT, heart-healthy parent wild-type; VAR, proband with *MYH6*-R443P.

Contractile data were collected from WT (heart healthy parent wild-type) and VAR (proband with the *MYH6*-R443P variant) iPSC-CMs at time points D20, D27, D34, and D41 (Supplementary Table S1). WT iPSC-CMs showed a higher percent shortening and relaxation rate at D20 compared to VAR. However, by D27, there were no significant differences in contractile parameters except values for contraction rate per minute (CPM), which was higher in WT iPSC-CMs (Supplementary Table S1). This indicates that the primary difference in contractile parameters between WT and VAR manifests at relatively early stages. Consequently, the focus of our study shifted to a detailed examination of D20 cells. At D20, the extent of shortening (μm), the % shortening, and the rates of shortening and relaxation were higher in WT iPSC-CMs in comparison with VAR (Table 2). In Figure 3A, the best-fit lines demonstrate the measurement of maximal cell shortening and relaxation rates. For this cell, we measured the following parameters: CPM (WT, 57.2 and VAR, 45.7), shortening (WT, 2.9 μm and VAR, 2.4 μm), percent shortening (WT, 6.7% and VAR, 4.3%), rate of shortening (WT, 28.3 μm/s and VAR 23.2 μm/s) and rate of relaxation (WT, 22.5 μm/s and VAR, 17.4 μm/s). Addition of 1 μM isoproterenol had no effect on iPSC-CMs mechanics of either cell type. However, both cell types responded to 1 Hz stimulation, contracting at 60 ± 4 (WT) and 64 ± 4 (VAR), and while CPM increased further with 2 Hz stimulation, neither cell type was able to respond at 2 Hz (WT, 73 ± 6 and VAR, 76 ± 8). The amplitude of the Ca^2+^ transient was higher in WT compared to the VAR (Figure 3B) cells, but no differences were observed in the rate of rise or fall of the transient (Supplementary Table S2). Isoproterenol had no effect on the amplitude of the Ca^2+^ transient in either cell type but did increase the rate of rise and fall of the transient in the VAR (Supplementary Table S2). Figure 3C shows representative action potentials for WT and VAR at D20, followed by a 5 s recording from the same cell. The APD_90_ for these cells are 298 and 300 ms for WT and VAR, respectively.

**TABLE 2:** Summary of contractile differences between WT and VAR iPSC-CMs at D20.

	CPM	Shortening (μm)	Percentage of shortening (%)	Shortening rate (μm/s)	Relaxation rate (μm/s)
WT	47 ± 2	2.9 ± 0.2	8.1 ± 0.7	28.1 ± 2.5	19.7 ± 2.0
VAR *MYH6*-R443P	40 ± 2*	2.3 ± 0.2*	5.6 ± 0.5*	19.9 ± 1.7*	11.0 ± 0.9*

For CPM measurements, *n* = 36 and 35 for WT and VAR, respectively. Ten contractions for each cell were averaged to give a single value for the contractile parameter. Only cells shortening inward from both edges were analyzed for shortening, % shortening, and rates of shortening/relaxation. CPM, contraction per minute; WT, heart-healthy parent wild-type; VAR, proband with *MYH6*-R443P. Values are means ± SE. One-way ANOVA, *P < 0.05.

Contractile data revealed that *MYH6*-R443P variant-inserted iPSC-CMs contracted more slowly, while the % shortening as well as the rates of shortening and relaxation were not different between the WTcc vs. inserted +/VAR and inserted VAR/VAR iPSC-CMs (Table 3 and Supplementary Table S3). All three of the CRISPRed genotypes responded to 1 and 2 Hz stimulation, but the inserted VAR/VAR iPSC-CMs had a higher CPM compared to WTcc at 2 Hz (Supplementary Table S3). The inserted VAR/VAR iPSC-CMs also had a greater shortening rate compared to the inserted +/VAR iPSC-CMs at 1 Hz but not 2 Hz. There were no differences in the Ca^2+^ transients of the WTcc, inserted +/VAR and inserted VAR/VAR iPSC-CMs (Supplementary Table S4). The mean action potential characteristics for the WT, VAR, WTcc, inserted +/VAR and inserted VAR/VAR were not different (Table 4). This is important as it demonstrates that neither the variant nor the CRISPR technology altered the electrical properties of the sarcolemma membrane.

**TABLE 3:** Summary of contractile differences between WTcc and VAR-inserted iPSC-CMs at D20.

	*n*	CPM	Shortening (μm)	Percentage of shortening (%)	Shortening rate (μm/s)	Relaxation rate (μm/s)
WTcc	60	66 ± 6	1.9 ± 0.09	5.1 ± 0.34	16.8 ± 1.23	10.9 ± 0.59
Inserted +/VAR	54	34 ± 2^∗^	1.8 ± 0.12^†^	4.6 ± 0.35	15.3 ± 1.04	11.6 ± 0.70
Inserted VAR/VAR	59	37 ± 2^∗^	2.2 ± 0.14	4.2 ± 0.27	19.1 ± 1.50	13.0 ± 1.05

Ten contractions were averaged for each cell (n). Only cells shortening inward from both edges were analyzed. CPM, contraction per minute; WTcc, CRISPRed control wild-type; +/VAR, *MYH6*-R443P heterozygous inserted; VAR/VAR, *MYH6*-R443P homozygous inserted. Values are means ± SE. One-way ANOVA, *P* < 0.05. *Denotes significant difference from WTcc. ^†^Denotes significant difference from Inserted VAR/VAR.

**TABLE 4:** Summary of action potentials at D20.

	*n*	Diastolic potential (mV)	Threshold potential (mV)	APD_90_ (ms)	Spike potential (mV)
WT	37	−40 ± 1	−34 ± 1	269 ± 15	19 ± 2
VAR *MYH6-*R443P	37	−37 ± 2	−34 ± 2	258 ± 8	16 ± 1
WT_CC_	10	−38 ± 2	−34 ± 3	269 ± 23	20 ± 2
Inserted +/VAR	24	−31 ± 2	−29 ± 2	337 ± 17	18 ± 2
Inserted VAR/VAR	14	−35 ± 4	−32 ± 4	315 ± 24	27 ± 4

*Each iPSC-CM’s diastolic potential, threshold potential, APD_90_, and spike potential were determined from the average of five different action potentials. APD_90_, time in ms for potential to return to 10% of initial (i.e., most negative) potential. WT, heart-healthy parent wild-type; VAR, proband with *MYH6*-R443P; WTcc, CRISPRed control wild-type; +/VAR, *MYH6*-R443P heterozygous inserted; VAR/VAR, *MYH6*-R443P homozygous inserted. Values are means ± SE. One-way ANOVA, *P* < 0.05.*

**FIGURE 4:**
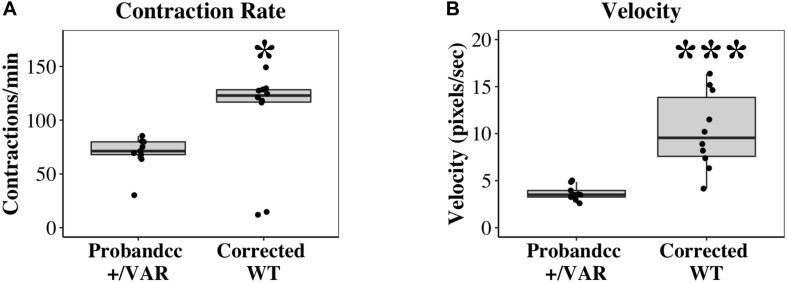
Contractile differences between Probandcc and corrected WT iPSC-CMs at D20-D22. **(A)** Contraction rate and **(B)** Velocity of iPSC-CMs were recorded at D20-D22 using an automated contractility software with four technical replicates for each genotype (Probandcc *n* = 9 and corrected WT *n* = 10). Probandcc, CRISPRed control proband; corrected WT, CRISPRed and corrected *MYH6*-R443P variant. The lines in the box plots indicate median, with maximum and minimum calculated values shown as whiskers. Each dot is a technical replicate. Wilcoxon signed-rank test, *P < 0.05 and ***P < 0.001.

In mass cell cultures, using a separate automated contractility software (see section “Materials and Methods” for details), we also analyzed velocity of corrected WT iPSC-CMs at D20-D22. With this analysis, we obtained similar results as single-cell studies. Corrected WT iPSC-CMs exhibit faster contraction (Figure 4A, *P* < 0.05) and increased velocity (Figure 4B, *P* < 0.0001) compared to Probandcc iPSC-CMs.

### Protein Expression of Typical Muscle Proteins

**FIGURE 5:**
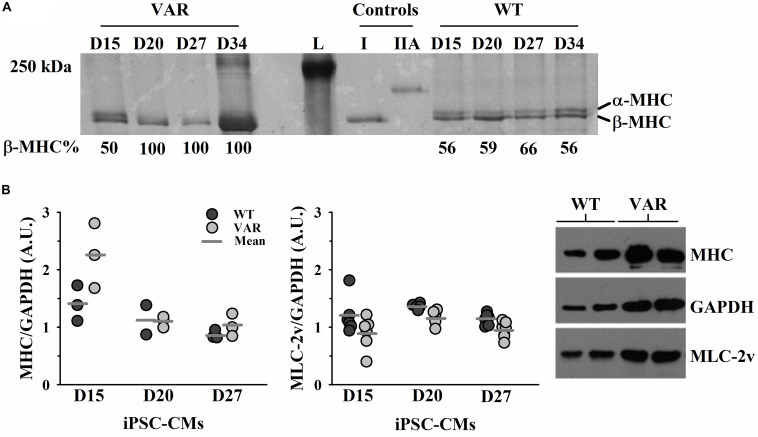
Upregulation of β-MHC in VAR at D15-D34. **(A)** Silver stained 5% SDS PAGE gel with each lane corresponding to the differentiation days and % of β-MHC expression listed beneath, as determined by densitometric analysis. **(B)** Protein expression in WT and VAR at D15, D20 and D27, as determined by densitometric analysis. Left: WT and VAR expression of MHC (*left*) and MLC2v (*right*), normalized to GAPDH. Values are means ± SEM of three technical replicates for each day and cell type. Right: Representative Western blot showing MHC, MLC2v, and GAPDH expression in WT and VAR at D20. L, ladder; I, type I skeletal muscle fiber; IIA, type IIA skeletal muscle fiber; WT, heart-healthy parent wild-type; VAR, proband with *MYH6*-R443P.

Both α-MHC and β-MHC were present in WT iPSC-CMs throughout the time course of D15-D34, while VAR iPSC-CMs showed the presence of only β-MHC after D15 (Figure 5A). The 12% SDS gel (Supplementary Figure S2) shows that both cell types contain multiple proteins known to exist in cardiac muscle, including actin and MLC2v. Western blot analyses confirmed the presence of MHC and MLC2v in all iPSC-CM samples and densitometric analyses found no difference between cell types in the expression of either total MHC (both α- and β- isoforms of MHC) or MLC2v (Figure 5B).

### Sarcomere Organization in Patient Cardiac Tissues

**FIGURE 6:**
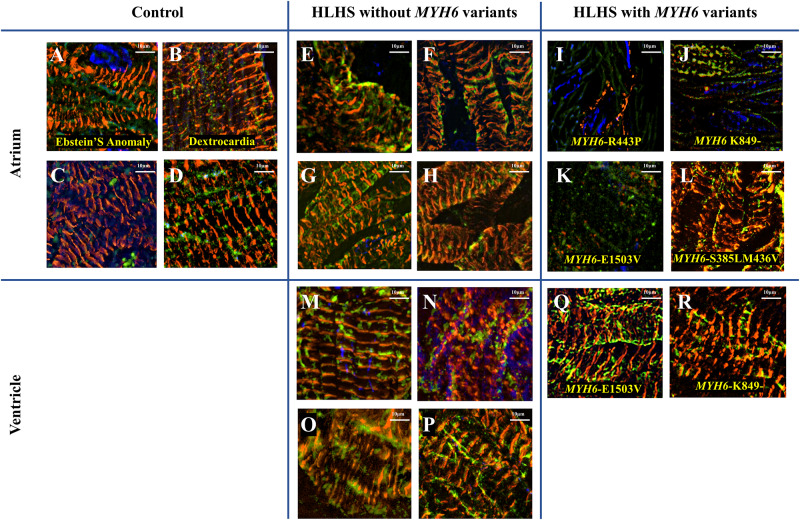
Dysmorphic sarcomeres were observed in atrial tissues from HLHS patients with *MYH6* variants, but not in ventricles. Sarcomeric α-actinin is in red, MF20 in green and dapi in blue. **(A–D)** Atrial tissues from HLHS free patients without *MYH6* variants. **(E–H)** Atrial tissues from HLHS patients without *MYH6* variants. **(I–L)** Atrial tissues from HLHS patients with *MYH6* variants. **(M–P)** Ventricular tissues from HLHS patients without *MYH6* variants. **(Q,R)** Ventricular tissues from HLHS patients with *MYH6* variants.

We observed a dysmorphic sarcomere phenotype in cardiac tissues from HLHS subjects with *MYH6* variants. Detailed sample information is listed in Table 5 and Supplementary Table S5. We also identified combined annotation dependent depletion (CADD) scores for variant pathogenicity for all the *MYH6* variants (GRCh38-v1.6 for SNP and GRCh38-v1.3 for deletion): 26.5 for *MYH6*-R443P (Figure 6I), 25.9 for *MYH6*-K849- (Figure 6J), 33 for *MYH6*-E1503V (Figure 6K), and 22.9 for *MYH6*-S385L/25.2 for M436V compound heterozygous variant (Figure 6L; Rentzsch et al., 2019).

**TABLE 5:** Tissue characteristics for Figure 6.

Sample label	Tissue source	Patient age (years)	Clinical diagnosis	*MYH6* genotype	Transplantation age
A	Left atrium	1 – 5	Ebstein’s anomaly	WT	N/A
B	Left atrium	1 – 5	Dextrocardia	WT	N/A
C	Inter atrial septum	20 – 25	Structurally normal	WT	N/A
D	Left atrium	1 – 5	Structurally normal	WT	N/A
E	Left atrium	10 - 15	HLHS	WT	At the same time of tissue sage
F	Left atrium	1 – 5	HLHS	WT	At the same time of tissue sage
G	Right atrium	1 – 5	HLHS	WT	N/A
H	Atrium (unknown side)	1 – 5	HLHS	WT	N/A
I	Left atrium	1 – 5	HLHS	R443P	N/A
J	Left atrium	1 – 5	HLHS	K849-	At the same time of tissue sage
K	Left atrium	10 - 15	HLHS	E1503V	At the same time of tissue sage
L	Atrial septum	1 – 5	HLHS	S385L and M436V	N/A
M	Ventricle	10 – 15	HLHS	WT	At the same time of tissue sage
N	Right ventricle	0 – 1	HLHS	WT	N/A
O	Ventricle	10 – 15	HLHS	WT	N/A
P	Right ventricle	0 – 1	HLHS	WT	N/A
Q	Right ventricle	10 – 15	HLHS	E1503V	Later than tissue age
R	Apex	1 – 5	HLHS	K849-	At the same time of tissue sage

Sarcomeres in the atria and ventricles of healthy (Figures 6A–D) and HLHS tissues without *MYH6* variants (Figures 6E–H and Supplementary Figures S4A–D,G) were organized except two samples, which are further described below. This was also the case in atrial tissues of other CHD subjects, including Ebstein’s Anomaly (Figure 6A) and Dextrocardia (Figure 6B). The organized sarcomere structure in HLHS patients without the *MYH6* variant is clearly seen by comparing the atrial tissue in Figure 6E with the ventricular tissue in Figure 6M, both obtained at the time of surgery from the same HLHS subject. Notably, 3 of the 4 atrial tissues from HLHS subjects possessing the *MYH6* variant (Figures 6I–L) exhibited poor sarcomere organization, while ventricular tissues showed normal structure (Figures 6Q,R). Sarcomeres in atrial tissues from HLHS subjects with *MYH6* variants are absent, or if detectable are disorganized [Figure 6I (*MYH6*-R443P), Figure 6J (*MYH6*-K849-), Figure 6K (*MYH6*-E1503V)]. The *MYH6*-R443P variant is in the head domain whereas *MYH6*-K849- and *MYH6*-E1503V are in the tail domain. The atrial and the ventricular tissues in Figures 6J,R are from a HLHS patient with *MYH6*- K849-, while the atrial and the ventricular tissues in Figures 6K,Q are from a HLHS patient with *MYH6*-E1503V. At the neonatal stage, sarcomeres are organized in both atrial tissues from HLHS patients with and without *MYH6* variants (Supplementary Figure S3). The atrial restriction of this sarcomere phenotype corresponds to the predominance of *MYH6* expression in the atria after the neonatal stage. The samples in Supplementary Figure S3B were from the same HLHS patient with *MYH6*-R443P (Figure 6I). Additional samples exhibited disrupted sarcomeres in HLHS subjects without an *MYH6* variant, one carrying a *Dystrophin* hemizygous variant (*DMD*-I228N, scaled CADD-score 26.6) (Supplementary Figure S4E) while the other HLHS subject did not reveal any sarcomere gene variants from whole exome sequencing (Supplementary Figure S4F).

## Discussion

HLHS etiology remains incomplete, in part due to its diverse phenotype and complex genetic inheritance, as well as environmental factors. To understand the molecular and cellular mechanisms of HLHS, we investigated patient-specific HLHS etiology using an iPSC-CM model and examined cardiac tissue from HLHS patients with sarcomere immunostaining. To further demonstrate the impact of the *MYH6*-R443P variant on HLHS, we used CRISPR/Cas9 gene editing to generate isogenic controls. Insertion of the *MYH6*-R443P variant into the heart healthy parent’s iPSCs recapitulated the dysmorphic sarcomere phenotype (Figure 1) and contractile dysfunction (compare Tables 2, 3). Correcting the *MYH6*-R443P variant in the proband’s iPSCs rescued the phenotypes of decreased CM differentiation efficiency, dysmorphic sarcomeres, and abnormal CPM and velocity (Figure 4).

Based on functional analysis of iPSC-CMs with the *MYH6*-R443P variant, the primary contractile defects were reduced CPM, impaired cell shortening (expressed as a % of cell length), and decreased rates of shortening and relaxation. These defects occurred early (by D20) and likely resulted from reduced development of the SR including the SERCA pump and the increased expression of the slow β-MHC isoform in the variant iPSC-CMs (Figure 5A). The reduced SR SERCA pump activity would explain the slower relaxation rates in the VAR. Regarding contraction rate, the β-MHC isoform is known to have a lower ATPase activity which limits cross-bridge cycle speed, and skeletal fibers containing this isoform are 3–4 times slower than α-MHC fast fibers (Schluter and Fitts, 1994; Fitts, 2008). The reduced contractile function in the *MYH6*-R443P variant compared to WT iPSC-CMs at D20 may be due to delayed development, as by D27 there were no differences in the contractility between the WT and VAR. Beyond D27, the loss of difference between cell types in contractile function was likely at least in part due to delayed development of the VAR particularly the SR. This is best demonstrated by the relaxation rate where at D20 the WT was twofold higher but by D27 the VAR relaxation rate had increased to match the WT (Supplementary Table S1). Despite no differences in shortening rate between WT and VAR past D20, the β-MHC content remained high in the VAR from D21 through D34 (Figure 5). We know iPSC-CMs are immature at D20, which may explain why cell contractility was not increased by adrenergic stimulation with isoproterenol (Supplementary Table S2). This lack of response most likely indicates immaturity of the sarcoplasmic reticulum, but also could indicate lack of the ß1 receptor on the surface membrane (Pillekamp et al., 2012).

The *MYH6*-R443P variant showed a reduced amplitude of the Ca^2+^ transients upon activation compared to WT, but both showed diminished Ca^2+^ release compared to adult rat ventricular cardiac cells (Wang and Fitts, 2017). It is likely that the reduced Ca^2+^ release relates to lower sarcoplasmic reticulum content and not to differences in surface membrane activation, as the recorded action potentials were identical between the WT and VAR. Differences in membrane excitability at D20 were noted, indicated by lower CPM in the VAR compared to the WT. Since the lowest membrane potential (referred to as diastolic potential) and the threshold potential were not different between cell types, this suggests that the rate of spontaneous depolarization to threshold may have been faster in the WT. However, our recordings did not show an obvious difference in the rate of depolarization to threshold between cell types (Figure 3 and Table 4). This may be because the difference is too small for detection during the single-cell level experiments performed at D20. Sorting out differences between the WT and VAR iPSC-CMs will require additional studies of organelle function (particularly the sarcoplasmic reticulum and myofilaments).

The basic contractile unit of striated (cardiac and skeletal) muscle is the sarcomere, a highly ordered structure containing contractile and regulatory proteins. Disruptions in sarcomere proteins have been implicated in many cardiac diseases (Redwood et al., 1999; Seidman and Seidman, 2001; Richard et al., 2003; Laing and Nowak, 2005). Here, we observed dysmorphic sarcomeres in tissues from HLHS patients with certain *MYH6* variants and these changes were phenocopied in iPSC-CMs generated from a HLHS patient with the *MYH6*-R443P variant. Sarcomere disorganization in cardiac tissues from three out of four HLHS patients with *MYH6* variants was observed in atrial but not in ventricular tissues (Figure 6). This result was expected as α-MHC protein expression is predominantly in the postnatal atria in humans. Likely, the *in vivo* contractile dysfunction is mainly in the atria, with the hypoplastic ventricular phenotype representing a developmental response resultant from diminished inflow pulsatility. Adding to the idea that poorly organized sarcomeres might contribute to the development of HLHS, atrial tissues from a HLHS patient with a costameric variant in *Dystrophin* (*DMD*) also showed sarcomere disruption (Supplementary Figure S4E). Furthermore, we observed that variants with the highest scaled CADD-scores: *MYH6*-R443P (26.5), *MYH6*-K849- (25.9), *MYH6*-E1503V (33) and *DMD*-I288N (26.6) had the most apparent disruptions in sarcomere structure in atrial tissues *ex vivo*. One limitation was lack of paired controls, however tissue samples showed convincing evidence of abnormalities (Figure 6).

Investigating HLHS etiology requires a physiological system of electrically and mechanically connected iPSC-CMs. One concern with employing iPSCs is the clonal variability; to reduce variability, we conducted all experiments multiple times (as indicated in each figure legend) in a non-biased, blinded fashion. A WT line was always differentiated along with experimental lines to compare differences among cell types at each differentiation stage. Additionally, isogenic *MYH6*-R443P cell lines were created using CRISPR/Cas9 gene editing and these lines recapitulated the phenotypes of patient-specific *MYH6*-R443P iPSC-CMs. An additional limitation is the heterogenous population of iPSC-CMs, including both atrial and ventricular cells. There are MLC-2a, MLC-2v, as well as double-positive iPSC-CMs in our cultures upon immunostaining at D31-D35 (data not shown). As our protein expression (Figure 5) and automated contractility assays (Figure 4) were analyzed in masse, they may underestimate the true heterogeneity of our cells. While we have a relatively pure population of cTnT positive CMs that express our cardiac gene of interest, *MYH6*, single-cell analyses similar to those completed by other groups may help further clarify their composition (Weber et al., 2016).

Despite being relatively immature, iPSC-CMs expressing the *MYH6*-R443P allele showed morphological and physiological differences from WT. iPSC-CMs could be used for clinical applications such as pharmacological drug testing, but these applications require mature CMs. A number of strategies have been employed to promote the maturity of iPSC-CMs, including: the administration of biochemical agents [adrenergic receptor agonists, Triiodothyronine (Yang et al., 2014b; Jackman et al., 2018), insulin-like growth factor 1 or microRNA (mir1 or mir208)], altering substrate stiffness or utilizing molds for cell-patterning (Ribeiro et al., 2015, 2016, 2017; Carson et al., 2016; Jung et al., 2016), application of mechanical stimuli (Hirt et al., 2014), and co-culture with different cell types to mimic human tissue, including bioengineering of 3D tissue (Yang et al., 2014a; Ronaldson-Bouchard et al., 2018). iPSC-CM maturation can then be confirmed using a multiparametric quality assessment, including gene expression profiling and structural, electrophysiological, and contractile measurements (Sheehy et al., 2014).

We showed contractile and sarcomere organization differences between WT and *MYH6*-R443P iPSC-CMs, however, how the early stage CM-phenotype affects cardiac morphogenesis *in vivo* and how this phenotype progresses to adulthood is complex and not understood. To investigate this progression *in vivo* it may be beneficial to use an animal model in parallel with iPSC-CMs. While murine models have opposite chamber-specific expression of *Myh6* and *Myh7*, zebrafish may be a suitable alternative for studying *MYH6*-associated CHD despite having a two-chambered heart. Importantly, zebrafish chamber-specific MHC expression is the same as humans. The phenotype of weak atrium (*wea*) *myh6*-mutant zebrafish exhibits a hypoplastic ventricle (Berdougo et al., 2003).

Our study proposes a new potential mechanism for the development of HLHS related to *MYH6*-R443P: sarcomere disorganization likely causes decreased atrial contractility and results in hypoplastic left ventricular development. The failing structural and contractile changes of the atrial sarcomere may effect hemodynamic changes that prevent the development of a normal left ventricle and downstream structures including the mitral valve, the aortic valve and the aorta. In addition, postnatally, atrial dysfunction due to sarcomere disorganization in single ventricle patients may result in decreased atrial kick, which would be expected to lead to heart failure over time. Further studies are needed to test potential ways to correct these contractile changes. It is possible that drugs targeting contractility may be able to alleviate some of the contractile changes due to the *MYH6*-R443P variant. Ultimately, we may be able to alter the developmental pathways and minimize or prevent the development of *MYH6* variant associated HLHS.

## Data Availability Statement

All datasets generated for this study are included in the article/Supplementary Material. Additional raw data will be made available by the authors, without undue reservation, to any qualified researcher.

## Ethics Statement

This study is in accordance with the principles outlined in the Declaration of Helsinki and institutionally approved research (IRB) protocols by the Children’s Hospital of Wisconsin (CHW, Milwaukee, WI, United States). Subjects were consented through the CHD Tissue Bank (IRB #CHW 06/229, GC 300) and the Wisconsin Pediatric Cardiac Registry (IRB #CHW 09/91, GC 889), IRB-approved research databases housed at CHW prior to inclusion in the study. Both biorepositories provided all cardiac tissue as well as iPSCs, from patients and family members, with associated clinical outcome variables.

## Author Contributions

AT-M and RF designed and directed the project. AT-M, RF, MEM, JWL, and AG were involved in planning and supervising the work. M-SK, RF, BF, JL, MC, MA, SS, LK, LT, and MM processed the experimental data, performed the analysis, and interpreted the data. M-SK took the lead in writing manuscript with support from AT-M, RF, BF, JL, LK, LT, MM, MA, and SS. All authors discussed the results and commented on the manuscript.

## Conflict of Interest

AT-M and MEM are cofounders of TAI Diagnostics (Milwaukee, WI, United States), a biotechnology company involved in transplant diagnostics, and members of its scientific advisory board. The remaining authors declare that the research was conducted in the absence of any commercial or financial relationships that could be construed as a potential conflict of interest.

## Footnotes

1 https://www.pulsevideoanalysis.com/
