# Supplementary material for: Contractility of Induced Pluripotent Stem Cell-Cardiomyocytes With an MYH6 Head Domain Variant Associated With Hypoplastic Left Heart Syndrome
Source: Front Cell Dev Biol. 2020 Jun 23;8:440. doi: 10.3389/fcell.2020.00440 (PMC7324479; doi:10.3389/fcell.2020.00440)
Supplement: Supplementary file 1 [file Data_Sheet_1.pdf]

## Supplementary Material

### Table of Contents


Supplemental Tables and Figures

Table S1: Summary of contractile differences between WT and VAR iPSC-CMs at D20-D41.

Table S2: Calcium transients of WT and VAR iPSC-CMs at D20.

Table S3: Summary of contractile differences between WTcc and *MYH6*-R443P inserted iPSC-CMs.

Table S4: Calcium transients of WTcc and VAR-inserted iPSC-CMs at D20.

Table S5: Tissue characteristics for Fig S4.

Figure S1: *MYH7* sequence at the homologous *MYH6*-R443P locus.

Figure S2: Actin and MLC-2v expression in WT and VAR iPSC-CMs.

Figure S3: Organized sarcomeres in neonatal (4-day-old) atrial tissues from HLHS patients.

Figure S4: Sarcomere structure in atrial and ventricular tissues from HLHS subjects without *MYH6* variants.

**Table S1.** Summary of contractile differences between WT and VAR iPSC-CMs at D20-D41.

| | Cell Age (Days) | *n* | CPM | *n* | Shortening (µm) | Percent Shortening (%) | Shortening Rate (µm/s) | Relaxation Rate (µm/s) |
|---|---|---|---|---|---|---|---|---|
| WT | D20 | 36 | 47 ± 2 | 33 | 2.9 ± 0.2 | 8.0 ± 0.7 | 28.1 ± 2.5 | 19.7 ± 2.0 |
| | D27 | 30 | 60 ± 2 | 29 | 2.2 ± 0.1 | 5.8 ± 0.5 | 22.9 ± 1.8 | 18.5 ± 1.6 |
| | D34 | 29 | 53 ± 4 | 25 | 2.1 ± 0.2 | 5.6 ± 0.6 | 23.9 ± 2.7 | 17.4 ± 1.4 |
| | D41 | 30 | 47 ± 3 | 26 | 2.0 ± 0.2 | 5.5 ± 0.6 | 21.2 ± 2.4 | 16.5 ± 1.6 |
| VAR *MYH6*-R443P | D20 | 35 | 40 ± 2 | 30 | 2.26 ± 0.2 | 5.6 ± 0.5* | 19.9 ± 1.7 | 11.0± 0.9* |
| | D27 | 22 | 38 ± 3* | 20 | 2.10 ± 0.2 | 6.5 ± 0.6 | 23.7 ± 1.9 | 20.7 ± 3.3 |
| | D34 | 30 | 49 ± 2 | 30 | 2.01 ± 0.2 | 5.9 ± 0.5 | 25.1 ± 2.6 | 16.4 ± 1.6 |
| | D41 | 27 | 43 ± 2 | 26 | 1.93 ± 0.2 | 5.0 ± 0.4 | 21.4 ± 1.7 | 15.3 ± 1.8 |

Ten contractions for each iPSC-CM were averaged to give a single value for each contractile parameter. Only cells shortening inward from both edges were analyzed for shortening and relaxation rates. CPM, contractions per minute; WT, heart-healthy parent wild-type; VAR, proband with MYH6-R443P. Values are means ± SE. Two-way ANOVA, * denotes within a given parameter, a difference from WT at the particular day. *$P < 0.05$.

**Table S2**. Calcium transients of WT and VAR iPSC-CMs at D20.

| | | *n* | Amplitude (ratio units) | Rate of Rise (ratio units/sec) | Rate of Fall (ratio units/sec) |
|---|---|---|---|---|---|
| WT | Untreated | 44 | 0.041 ± 0.006 | 0.559 ± 0.054 | 0.169 ± 0.022 |
| VAR *MYH6*-R443P | Untreated | 41 | 0.021 ± 0.0002† | 0.443 ± 0.038 | 0.122 ± 0.019 |
| WT | No Isoproterenol | 15 | 0.055 ± 0.012 | 0.733 ± 0.096 | 0.246 ± 0.049 |
| | Isoproterenol | 6 | 0.049 ± 0.003 | 0.920 ± 0.035 | 0.245 ± 0.014 |
| VAR *MYH6*-R443P | No Isoproterenol | 18 | 0.019 ± 0.001 | 0.313 ± 0.023 | 0.120 ± 0.013 |
| | Isoproterenol | 11 | 0.022 ± 0.003 | 0.426 ± 0.045* | 0.201 ± 0.046* |

Ten $Ca^{2+}$ transients were averaged for each cell type (n). WT, heart-healthy parent wild-type; VAR, proband with *MYH6*-R443P. Values are means ± SE. †Denotes difference from WT cells. *Denotes difference from untreated cells. One-way ANOVA, † and * $P < 0.05$.

**Table S3**. Summary of contractile differences between WTcc and *MYH6*-R443P inserted iPSC-CMs at D20.

| | Frequency | n | CPM | Shortening (μm) | Percent Shortening (%) | Shortening Rate (μm/s) | Relaxation Rate (μm/s) |
|---|---|---|---|---|---|---|---|
| WTcc | Endogenous | 5 | 49 ± 21 | 2.1 ± 0.3 | 5.0 ± 1.0 | 17.8 ± 2.4 | 10.3 ± 1.2 |
| | 1 Hz | 5 | 88 ± 13 | 1.8 ± 0.3 | 4.3 ± 0.8 | 13.7 ± 1.4 | 10.4 ± 1.3 |
| | 2 Hz | 5 | 124 ± 2 | 1.2 ± 0.1 | 2.9 ± 0.5 | 10.6 ± 1.1 | 8.6 ± 0.4 |
| Inserted +/VAR | Endogenous | 5 | 30 ± 6 | 1.5 ± 0.2† | 4.4 ± 0.8 | 11.6 ± 1.1† | 10.0 ± 1.2 |
| | 1 Hz | 5 | 74 ± 3 | 1.4 ± 0.2 | 4.2 ± 0.7 | 11.0 ± 1.2† | 9.74 ± 1.1 |
| | 2 Hz | 5 | 127 ± 1 | 1.0 ± 0.2 | 3.1 ± 0.5 | 9.2 ± 1.1 | 9.4 ± 1.0 |
| Inserted VAR/VAR | Endogenous | 6 | 43 ± 5 | 3.4 ± 0.4 | 5.0 ± 0.6 | 31.7 ± 4.0 | 11.9 ± 1.6 |
| | 1 Hz | 6 | 90 ± 2 | 2.5 ± 0.3 | 3.9 ± 0.7 | 25.4 ± 2.8 | 21.0 ± 3.9 |
| | 2 Hz | 6 | 144 ± 1* | 1.5 ± 0.3 | 2.5 ± 0.6 | 18.1 ± 3.2 | 16.2 ± 1.6 |

For each iPSC-CM (n), ten contractions were averaged to give a single value for each contractile variable. Only cells shortening inward from both edges were analyzed. CPM, contractions per minute; WTcc, CRISPRed control wild type; +/VAR, *MYH6*-R443P heterozygous inserted; VAR/VAR, *MYH6*-R443P homozygous inserted. Values are means ± SE. *Denotes difference from WT. †Denotes difference between heterozygous inserted and homozygous inserted iPSC-CMs. One-way ANOVA, † and * $P < 0.05$.

**Table S4.** Calcium transients of WTcc and VAR-inserted iPSC-CMs at D20.

| | *n* | Amplitude | Rate of Rise (ratio units/sec) | Rate of Fall (ratio units/sec) |
|---|---|---|---|---|
| WTcc | 19 | 0.033 ± 0.004 | 0.544 ± 0.061 | 0.136 ± 0.037 |
| Inserted +/VAR | 18 | 0.039 ± 0.003 | 0.446 ± 0.050 | 0.169 ± 0.038 |
| Inserted VAR/VAR | 8 | 0.038 ± 0.002 | 0.500 ± 0.030 | 0.081 ± 0.010 |

Ten transients were averaged for each iPSC-CM (n). WTcc, CRISPRed control wild type; +/VAR, *MYH6*-R443P heterozygous inserted; VAR/VAR, *MYH6*-R443P homozygous inserted. Values are means ± SE.

**Table S5.** Tissue characteristics for Figure S4.

| Sample Label | Tissue Source | Patient Age (years) | Clinical Diagnosis | *MYH6* Genotype | Transplantation age |
|---|---|---|---|---|---|
| A | Inter atrial septum | 1 - 5 | HLHS | WT | N/A |
| B | Atrium (unknown side) | 1 - 5 | HLHS | WT | N/A |
| C | Atrium (unknown side) | 1 - 5 | HLHS | WT | Later than tissue age |
| D | Inter atrial septum | 5 - 10 | HLHS | WT | N/A |
| E | Left atrium | 1 - 5 | HLHS | WT (*DMD*-I228N) | At the same time of tissue age |
| F | Left atrium | 10 - 15 | HLHS | WT | At the same time of tissue age |
| G | Left ventricle | 10 - 15 | HLHS | WT | At the same time of tissue age |

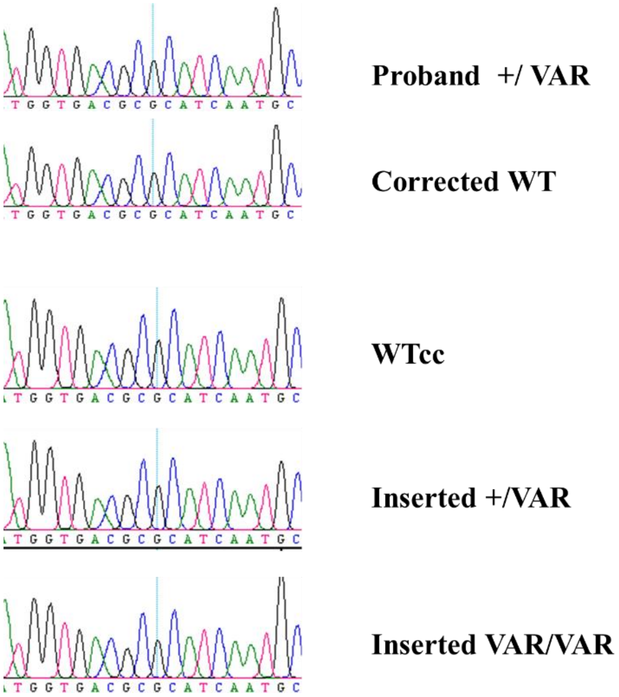


**Figure S1.** *MYH7* sequence at the homologous *MYH6*-R443P locus.

In inserted or corrected *MYH6*-R443P variant, CRISPR activity was specific for *MYH6* and not for *MYH7*. The PCR primers were 5’ gtctctcctccaccttcag3’ for forward and 5’tggtgttcttgttgggtgtg3’ for reverse, and the same primers were used for Sanger sequencing. WTcc, CRISPRed control wild type; +/VAR, *MYH6*-R443P heterozygous inserted; VAR/VAR, *MYH6*-R443P homozygous inserted; Probandcc, CRISPRed control proband; corrected WT, CRISPRed and corrected *MYH6*-R443P variant.

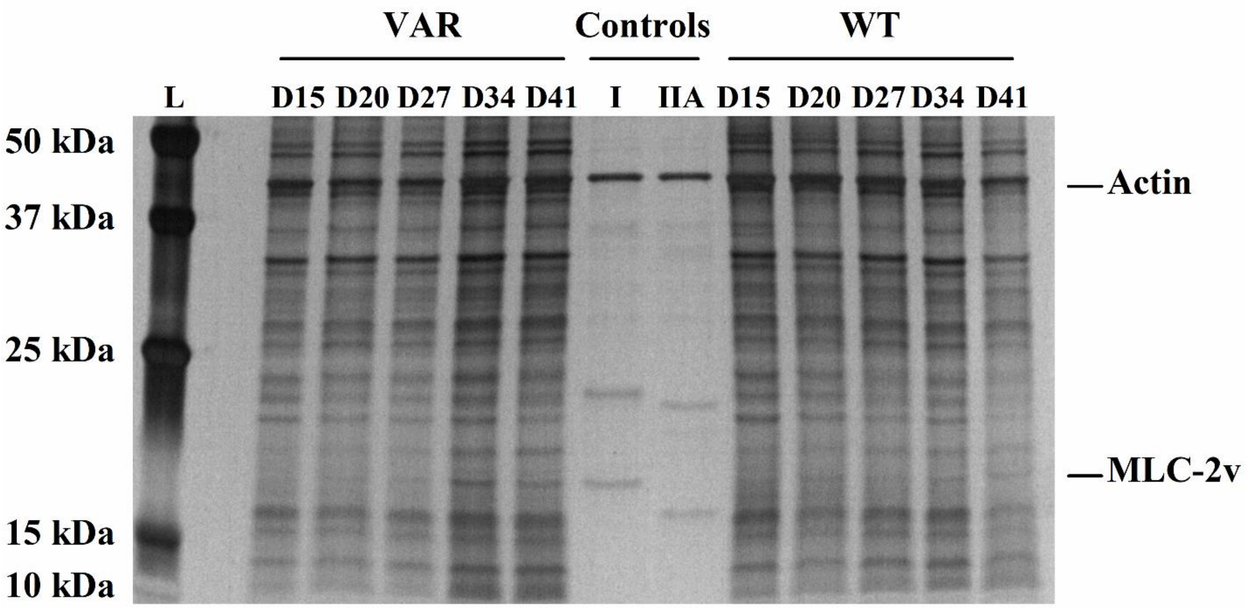


**Figure S2.** Actin and MLC-2v expression in WT and VAR iPSC-CMs.

Silver stained 12% SDS PAGE gel with each lane corresponding to the differentiation days. WT and VAR expressed actin and MLC2v at all days shown. L, ladder; I, type I skeletal muscle fiber; IIA, type IIA skeletal muscle fiber; WT, heart-healthy parent wild-type; VAR, proband with MYH6-R443P.

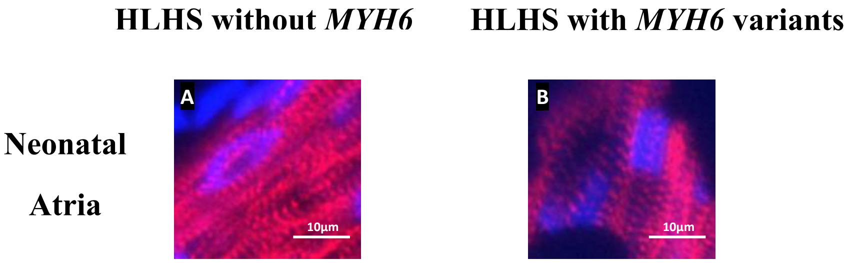


**Figure S3.** Organized sarcomeres in neonatal (4-day-old) atrial tissues from HLHS patients.

Sarcomeric α-actinin is in red and dapi in blue. **(A)** without an *MYH6* variant and **(B)** with an *MYH6*-R443P variant.

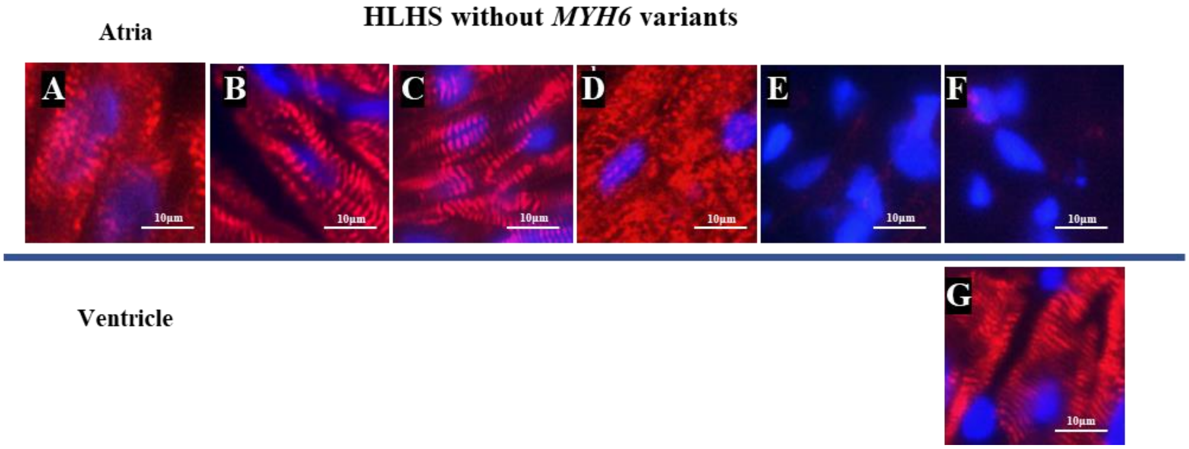


**Figure S4.** Sarcomere structure in atrial and ventricular tissues from HLHS subjects without *MYH6* variants.

Sarcomeric α-actinin is in red and dapi in blue. **(A-D)** Organized sarcomeres in atrial tissues. **(E)** Disrupted sarcomeres in atrial tissue from an HLHS subject with a Dystrophin (*DMD*) gene variant (*DMD*-I228N, CADD-score 26.6 (GRCh38-v1.6)) **(F)** Disrupted sarcomeres in atrial tissue from an HLHS subject with unknown variant. **(G)** Organized sarcomeres in ventricular tissue from the same HLHS subjects as **(F).**
